# Evaluating antibody functional activity and strain-specificity of vaccine candidates for malaria in pregnancy using in vitro phagocytosis assays

**DOI:** 10.1186/s13071-018-2653-7

**Published:** 2018-01-29

**Authors:** Mirja Hommel, Jo-Anne Chan, Alexandra J. Umbers, Christine Langer, Stephen J. Rogerson, Joseph D. Smith, James G. Beeson

**Affiliations:** 1 Burnet Institute, Melbourne, VIC Australia; 2 Department of Medicine, Peter Doherty Institute, University of Melbourne, Melbourne, VIC Australia; 3 Center for Infectious Diseases Research, Seattle, WA USA; 4 Department of Microbiology and Central Clinical School, Monash University, Clayton, VIC Australia

**Keywords:** Malaria in pregnancy, Placental malaria, Vaccines, Immunity, Opsonic phagocytosis

## Abstract

**Background:**

Malaria in pregnancy is a major cause of poor maternal and infant health, and is associated with the sequestration of *P. falciparum*-infected erythrocytes (IE) in the placenta. The leading vaccine candidate for pregnancy malaria, VAR2CSA, has been shown to induce antibodies that inhibit IE adhesion to the placental receptor chondroitin sulfate A (CSA), potentially preventing placental infection. However, the ability of vaccination-induced antibodies to promote opsonic phagocytosis is not well defined, but likely to be an important component of protective immunity.

**Methods:**

We investigated the use of an opsonic phagocytosis assay to evaluate antibodies induced by pregnancy malaria vaccine candidate antigens based on VAR2CSA. Opsonic phagocytosis was measured by flow cytometry and visualized by electron microscopy. We measured vaccine-induced antibody reactivity to placental type IEs from different geographical origins, and the functional ability of antibodies raised in immunized rabbits to induce phagocytosis by a human monocyte cell line.

**Results:**

Immunization-induced antibodies showed a mixture of strain-specific and cross-reactive antibody recognition of different placental-binding parasite lines. Antibodies generated against the DBL5 and DBL3 domains of VAR2CSA effectively promoted the opsonic phagocytosis of IEs by human monocytes; however, these functional antibodies were largely allele-specific and not cross-reactive. This has significant implications for the development of vaccines aiming to achieve a broad coverage against diverse parasite strains. Using competition ELISAs, we found that acquired human antibodies among pregnant women targeted both cross-reactive and allele-specific epitopes, consistent with what we observed with vaccine-induced antibodies.

**Conclusions:**

Vaccines based on domains of VAR2CSA induced opsonic phagocytosis of IEs in a strain-specific manner. Assays measuring this phagocytic activity have the potential to aid the development and evaluation of vaccines against malaria in pregnancy.

**Electronic supplementary material:**

The online version of this article (10.1186/s13071-018-2653-7) contains supplementary material, which is available to authorized users.

## Background

In malaria endemic areas, pregnant women are at high risk of placental malaria [1]. Despite recent gains in malaria control, approximately 125 million pregnancies remain at risk of malaria and adverse outcomes [2]. Thus, there is an urgent need for the development of effective malaria vaccines. In pregnancy, *Plasmodium falciparum*-infected erythrocytes (IEs) sequester in the placenta via adhesion to chondroitin sulphate A (CSA) [3]. This interaction is mediated by a specific variant of the *Plasmodium falciparum* erythrocyte membrane protein 1 (PfEMP1) family known as VAR2CSA, and possibly other interactions [4]. Although VAR2CSA shows high sequence diversity [5], some antibody epitopes in different domains are partially overlapping between VAR2CSA variants or have limited global antigenic diversity [6–8] and therefore support VAR2CSA as a potential vaccine candidate. Acquired antibodies directed against VAR2CSA are associated with the inhibition of CSA-binding by IEs [9, 10], reduced placental malaria [11] and improved pregnancy outcomes [12]. Funding has recently been secured for pre-clinical development and phase I trials of VAR2CSA-based vaccines.

To date, the focus of vaccine development in pregnancy malaria has been largely limited to generating antibodies that inhibit adhesion to CSA [13]. However, opsonizing antibodies to the DBL domains of VAR2CSA may also play an important role in protective immunity [14] by acting synergistically with binding-inhibition antibodies, or independently. It is known that cytophilic antibodies to VAR2CSA predominate in pregnancy [14, 15], and these can interact with Fc-receptors to promote the opsonic phagocytosis of IEs [16]. Acquired antibodies to CSA-binding isolates that express VAR2CSA promote opsonic phagocytosis and are associated with better pregnancy outcomes [17, 18]. Furthermore, VAR2CSA-specific monoclonal IgG1 antibodies isolated from malaria-exposed women can opsonize IEs expressing VAR2CSA for phagocytosis by monocytes [19]. Therefore, we investigated the potential use of an established high-throughput, low-cost opsonic phagocytosis assay for evaluating VAR2CSA vaccines. We used the DBL5 and DBL3 domains of VAR2CSA as our model vaccine antigens as VAR2CSA-specific antibodies acquired from placental infections appear to strongly target these domains [19–21]. Antibodies to both domains have been shown to partially inhibit IE adhesion to CSA [13, 14, 22], thus supporting the idea that these domains might be important targets of protective immunity. Other regions of VAR2CSA are also important targets and vaccine candidates [3], such as the N-terminal region (DBL1-DBL2, and ID1-DBL2X-ID2a), which is under development as a potential vaccine candidate [23–26].

## Methods

### Study design

To study the properties of vaccine-induced antibodies against pregnancy malaria, we measured the opsonic phagocytosis-inducing potential of antibodies to DBL5 and DBL3 raised through immunization of rabbits, which is a widely used pre-clinical model for malaria vaccine development. We quantified antibody recognition of the IE surface of placental-binding parasites and the level of opsonic phagocytosis activity using a human monocyte cell line, THP-1, which allowed for standardized assays [27]. Immunizations were performed as previously described, under ethics approval [28].

### Study participants

Pooled serum samples from malaria-exposed pregnant women residing in Madang, Papua New Guinea and Malawi [6] were used as positive controls. Sera from non-immune adult residents of Melbourne, Australia were used as negative controls.

### Materials

Briefly, DBL5 or DBL3 immunization of nine rabbits was performed using one of three recombinant proteins derived from IT4- (equivalent to parasite line CS2), 7G8- or 3D7 DBL5 alleles, as previously described [22]. Parasite isolates expressing VAR2CSA and derived from diverse geographical origins [CS2 (IT isolate, Brazil), HCS3 (Thailand), 3D7-CSA (3D7 reference isolate), XIE-CSA (Papua New Guinea) and 7G8-CSA (Peru)] were maintained and selected for the CSA-binding phenotype as described [6]. The expression of *var2csa* as the dominant *var.* gene in these isolates was described previously [6]. Isolate E8B (parental line of CS2 isolate) was used as a non-CSA binding control [7]. Rabbit antibodies were harvested pre-immunization (PI) and final bleed (FB) post-immunization. Analyses of overlap and differences between VAR2CSA sequences from the different isolates, and their relationship to global isolates, has been previously reported [6, 22].

### Immunoassays

IgG binding to the IE surface of CSA-binding isolates was measured by flow cytometry using previously established assays [6, 29]. Human immune sera or rabbit anti-DBL5 or anti-DBL3 polyclonal antibodies were tested for their ability to promote the opsonic phagocytosis of IEs using the monocytic cell line THP-1, as described in detail elsewhere [27, 29]. The level of phagocytic activity was measured by flow cytometry and quantified as the proportion of THP1 cells that had phagocytosed IEs (labelled with ethidium bromide); activity was then expressed as a percentage of the activity seen with a positive reference control (rabbit anti-human erythrocyte antibody or pooled serum from malaria-exposed pregnant women) [27, 29]. A pool of serum from residents of Melbourne, Australia, was used as a negative control in all assays. Phagocytosis of IEs by THP-1 monocytes was also visualized by scanning and transmission electron microscopy using standard techniques [29, 30]. *Plasmodium falciparum* was cultured in vitro in medium supplemented with 10% pooled human serum [6]. Statistics were performed using GraphPad Prism Software version 6.0. Competition ELISAs were used to evaluate cross-reactive and allele-specific antibodies to DBL5; to this end, serum samples were incubated with titrated amounts of the indicated DBL5 domain prior to running the ELISA. The serum samples were pre-incubated with titrated amounts (10, 5, 0.5 and 0.005 μg/ml) of 7G8- or 3D7-derived DBL5 for 1 h at room temperature. Pre-incubation with PBS was used as control. All sera were then tested for reactivity to DBL5 in ELISA at a final concentration of 1:250 and 50 μl were used per well. A selection of serum samples from malaria-exposed pregnant women resident in Malawi were used in these assays.

## Results

### Antibody levels and recognition of IEs

**Fig. 1:**
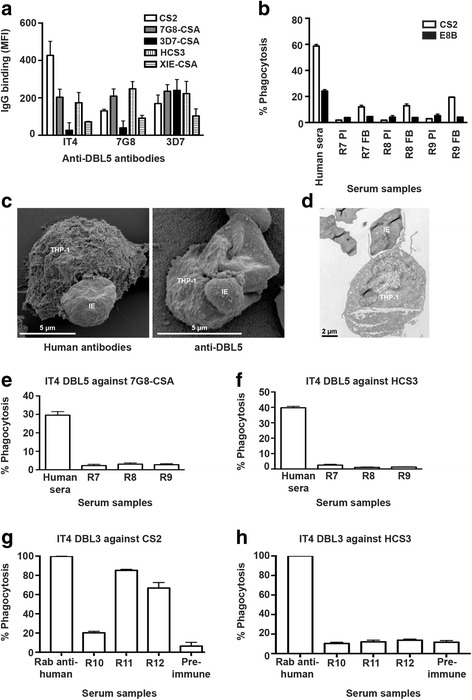
Specificity and functional activity of vaccine antibodies to the DBL5 and DBL3 domains of VAR2CSA. **a** The level of anti-DBL5 antibodies binding to the IE surface of different CSA-binding isolates (CS2, 7G8-CSA, 3D7-CSA, HCS3 and XIE-CSA) was measured by flow cytometry. Antibody reactivity varied depending on the recombinant proteins (derived from IT4, 7G8 or 3D7 DBL5 alleles) used for immunization. Bars represent mean and standard deviation of samples tested in duplicate; IgG binding levels are expressed as geometric mean fluorescence intensity (MFI). **b** Opsonic phagocytosis of *P. falciparum*-IEs by undifferentiated THP-1 cells using pre-immune (PI) and final bleed (FB) sera from immunized rabbits. Opsonic phagocytosis activity was significant for CS2 IEs (*P* = 0.024) but not for the isogenic control, non-CSA binding E8B parasite line (*P* = 0.74). Means and standard deviation of samples tested in duplicate are shown. Initial attachment and engulfment of CS2 IEs by THP-1 monocytes were visualized by scanning (**c**) and transmission electron microscopy (**d**). For scanning electron microscopy, IE were opsonized by a pool of immune human sera from pregnant Papua New Guinean women, or by polyclonal rabbit anti-DBL5 immune sera, while only the latter was used for transmission electron microscopy. **e**, **f** Opsonic phagocytosis of *P. falciparum*-IEs by undifferentiated THP-1 cells using final bleed sera from rabbits immunized with IT4 DBL5 (R7-R9). There was very little phagocytosis of 7G8-CSA (**e**) or HCS3 (**f**) parasites. A plasma pool from *P. falciparum* exposed pregnant women was used as a control in the phagocytosis assay. Means and standard deviation of samples tested in duplicate are shown. **g**, **h** Opsonic phagocytosis of *P. falciparum*-IEs by undifferentiated THP-1 cells using final bleed sera from rabbits immunized with IT4 DBL3 (R10-R12). Antibodies effectively promoted opsonic phagocytosis of CS2-IEs (**g**), but there was little phagocytosis of the heterologous isolate, HCS3 (**h**). A rabbit anti-human erythrocyte antibody was used as a positive control and the level of phagocytosis is expressed as a percentage of the positive control. Means and standard deviation of samples tested in duplicate are shown

Previous studies demonstrated that antibodies to DBL5 raised in rabbits reacted with recombinant proteins by ELISA [22]. IgG from rabbits immunized with different DBL5 alleles (IT4, 7G8 and 3D7) showed varied reactivity to native VAR2CSA expressed on the surface of IEs of different CSA-binding parasite lines when measured by flow cytometry (Fig. 1a), as reported previously [22]. IgG-binding showed a significant amount of strain-specificity and only partial cross-reactivity between different CSA-binding isolates that express VAR2CSA, despite there being substantial sequence identity between the different DBL5 alleles [21]. Total IgG binding to the surface of CS2 IEs was highest in sera from animals immunized with the DBL5 IT4 allele. IgG binding to 3D7-CSA IEs was highest in rabbits immunized with the DBL5 3D7 allele, but overall antibodies raised against the 3D7 allele showed broader reactivity across isolates. The DBL5 domain had been previously identified as an important target of naturally acquired antibodies in pregnant women [21].

### Vaccine antibodies promote opsonic phagocytosis of IEs expressing VAR2CSA

We tested rabbit antibodies for their ability to promote the opsonic phagocytosis of CS2 and E8B IEs (non-CSA binding line, same genotype as CS2). Antibodies generated by immunization induced significantly higher levels of opsonic phagocytosis of CS2 IEs compared to pre-immunization sera (Fig. 1b; *t*_(__2__)_ = 6.3, *P* = 0.024). However, there was very little opsonic phagocytosis with IEs of the E8B line, which does not express VAR2CSA or bind to CSA (Fig. 1b; *t*_(__2__)_ = 0.3, *P* = 0.74) [1]. This confirms that the opsonic phagocytosis mediated by anti-DBL5 antibodies is largely specific, identifying the potential use of this assay for VAR2CSA vaccine evaluation. The initial attachment and engulfment of CS2 IEs by THP-1 monocytes was visualized by scanning (Fig. 1c) and transmission (Fig. 1d) electron microscopy. Phagocytosis of IEs was observed with IEs opsonized with antibodies from malaria-exposed pregnant women from PNG or polyclonal rabbit anti-DBL5 antibodies. These images show, for the first time, processes from THP-1 cells attaching to IEs to initiate engulfment and further support data from flow cytometric analyses of phagocytosis.

### Opsonic phagocytosis activity of vaccine anti-VAR2CSA DBL5 antibodies is strain-specific

To investigate the strain-specificity or cross-reactivity of vaccine-induced opsonizing antibodies to placental-binding parasite isolates, we measured levels of phagocytosis induced by opsonization with the genetically distinct isolates 7G8-CSA (Fig. 1e) and HCS3 (Fig. 1f). Antibodies to IT4 DBL5 did not effectively promote substantial opsonic phagocytosis of either 7G8-CSA or HCS3 IEs. In contrast, sera from malaria-exposed Malawian pregnant women, used as a positive control, promoted high levels of phagocytosis with both parasite lines. This suggests that antibodies raised against the DBL5-IT4 variant do not cross-react well enough to promote sufficient opsonic phagocytosis in heterologous CSA-binding parasite isolates.

We extended these studies by testing antibodies raised against the DBL3 domain (IT4 allele). Antibodies to DBL3 also effectively promoted opsonic phagocytosis of the homologous isolate, CS2-IEs (Fig. 1g). However, as seen with DBL5 antibodies, there was little phagocytosis of a heterologous isolate, HCS3, indicating strain-specificity of these functional antibodies (Fig. 1h). These findings have significant implications for generating broadly reactive antibodies by vaccines based on VAR2CSA.

### Naturally acquired antibodies in pregnant women target variant-specific and cross-reactive antibodies on DBL5

**Fig. 2:**
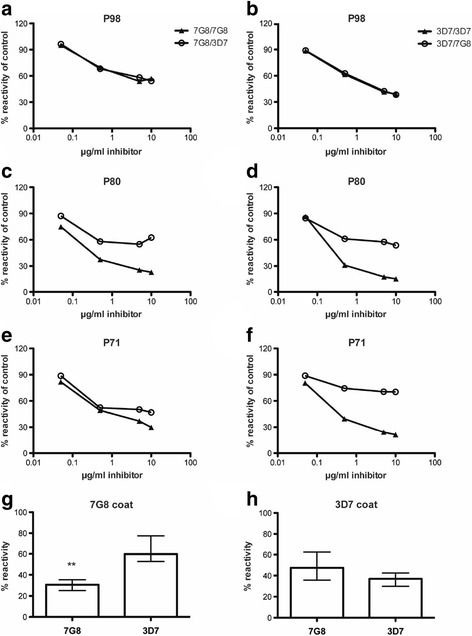
Naturally acquired antibody responses to DBL5 domain of VAR2CSA. A selection of sera from malaria-exposed Malawian women was tested for allele-specific or cross-reactive IgG binding to DBL5 in competition ELISA. Panels **a**-**f** show examples with cross-reactivity, allele-specificity, or a mixed pattern. Figures show the percent IgG reactivity with increasing concentration of competing DBL5 allele added. Panels **a**, **c**, **e** show results where plates were coated with 7G8 allele and either 7G8 or 3D7 alleles were used as the competitors. Panels **b**, **d**, and **f** show results using the 3D7 allele as the coating antigens. Median values (with IQR) of percentage allele specificity for all individuals (*n* = 10) tested are also shown in **g** and **h**. ** *P* = 0.0046

To further understand the allele-specific activity of antibodies to VAR2CSA domains, we evaluated antibodies from a selection (*n* = 10) of malaria-exposed pregnant women for cross-reactive and allele-specific antibodies to DBL5 using competition ELISAs. Samples that showed significant reactivity to the two alleles of DBL5 that were included in these assays. We evaluated the ability of the DBL5-3D7 to inhibit IgG binding to DBL5-7G8, and *vice versa* (Fig. 2). Overall, we found evidence of a mixture of cross-reactive and allele-specific antibodies, with substantial variation between individuals in the relative levels of cross-reactive or allele-specific antibodies. These findings are consistent with our observations that vaccine-induced antibodies comprise allele-specific and cross-reactive antibodies and that there are significant antigenic differences between DBL5 alleles. Because human antibodies are comprised of a mixture of antibodies to different VAR2CSA domains and alleles acquired through natural exposure, it is not possible to test these for allele-specificity in functional assays using intact IEs. Future studies using human monoclonal antibodies and/or domain-specific affinity-purified antibodies may be needed to investigate this further.

## Discussion

We conducted an exploratory study to evaluate the potential value of a high-throughput opsonic phagocytosis assay to measure functional immunity generated by candidate VAR2CSA vaccines. We used antibodies generated against the DBL5 domain, which is an important target of human antibodies, contains cross-reactive epitopes and antibodies against DBL5 have been shown to inhibit adhesion [13, 21, 22]. Studies were extended to antibodies against the DBL3 domain, which is also a target of acquired immunity [19] and antibodies to this domain can inhibit adhesion [13, 21]. Furthermore, prior studies have suggested that global diversity of DBL3 domains may be limited [6], which would be favorable for vaccine development. Our findings demonstrate that immunization with these recombinant VAR2CSA domains induced antibodies capable of opsonizing IEs for phagocytic clearance by monocytes, thus further supporting these domains for inclusion in a VAR2CSA-based vaccine. We also visualized the initial attachment and engulfment of IEs, opsonized by vaccine-induced antibodies using electron microscopy. Importantly, the functional activity of these vaccine-induced antibodies appears to have substantial strain-specificity, despite some level of cross-reactivity to native VAR2CSA on the surface of intact IEs being detected when quantified by flow cytometry. Our findings suggest that vaccination with a single VAR2CSA allele is unlikely to generate broadly reactive opsonizing antibodies that cover the global diversity present in VAR2CSA, which is consistent with previous findings of strain-specificity of human acquired antibodies [7]. Further studies are required to identify vaccine constructs, allele combinations, or approaches that will induce strain-transcendent activity. For example, an antigen derived from the N-terminal region (ID1-DBL2x) has generated cross-reactive antibodies that inhibit adhesion, and looks promising as a vaccine candidate [26].

Malaria-exposed multigravid women naturally acquire antibodies that promote the phagocytosis of CSA-binding IEs [31]. This immune response has been proposed to contribute to protection against maternal anemia [32] and low birth weight [18]. The capacity of these naturally acquired antibodies to promote opsonic phagocytosis of IEs has been validated using human immune sera [18, 31, 32]. Recent studies have also suggested that VAR2CSA is a major target of naturally-acquired human antibodies that promote the phagocytosis of CSA-binding IEs [14]. Here, we demonstrate a high throughput flow cytometry-based phagocytosis assay as an appropriate tool to evaluate antibody responses generated by candidate VAR2CSA vaccines and complement the more established adhesion-inhibition assay. Using this approach, we showed that a potential malaria vaccine candidate is able to elicit functional antibodies that promote the opsonic phagocytosis of placental-binding IEs. Furthermore, studies with *P. falciparum* isolates engineered to have reduced or absence PfEMP1 expression suggested that PfEMP1 is the dominant target of acquired antibodies to the IE surface among children, non-pregnant adults, and pregnant women, including antibodies that promote opsonic phagocytosis [29, 33].

We observed variable antibody recognition of different CSA-binding IE isolates by antibodies against different DBL5 alleles, suggesting a degree of cross-reactivity, but some strain-specificity, likely due to sequence diversity as noted previously [1, 7, 22, 28]. Despite the DBL5-IT4 immune sera showing moderate recognition of 7G8-CSA and HCS3 isolates by flow cytometry (in addition to high reactivity to CS2 IEs), this did not translate to functional antibody activity. Some recent data suggest that anti-DBL5 adhesion-blocking antibodies may be more strain-transcendent [34]. Our findings using competition ELISA with samples from malaria-exposed pregnant women also support the conclusion that antibodies to DBL5 are comprised of both strain-specific and cross-reactive antibodies. We chose the DBL5 domain as a model VAR2CSA vaccine candidate, and extended our findings to another important domain, DBL3. However, other domains may also induce opsonic phagocytosis [19] and a further detailed analysis of the cross-reactivity or strain-specificity of leading VAR2CSA vaccine candidates should be assessed. As this study aimed to establish a proof-of-principle, its scope was exploratory in nature and we did not perform an exhaustive analysis of all proposed vaccine constructs.

## Conclusions

Effective vaccine design aims for the generation of broadly cross-reactive antibodies to various isolates, and possibly antibodies with multiple functional activities. Vaccine development also requires the establishment of laboratory assays that can predict functional immunity. Findings presented here further support the use of phagocytosis assays as a complementary approach to assessing current and future vaccine candidates aimed at preventing malaria in pregnancy.

## Additional file

Additional file 1: Table S1. The raw data supporting Fig. 1. (XLSX 37 kb)

## Abbreviations

CSA: Chondroitin sulfate A
DBL5: Duffy binding-like domain 5
ELISA: Enzyme-linked immune-sorbent assay
IE: *Plasmodium falciparum*-infected erythrocytes
PfEMP1: *Plasmodium falciparum* erythrocyte membrane protein 1
PNG: Papua New Guinea
VAR2CSA: Variant surface antigen 2 against chondroitin sulfate A
